# Evidence Afforded by the Microscope in a Case of Rape

**Published:** 1858-08

**Authors:** 


### ECLECTIC DEPARTMENT,

### AND SPIRIT OF THE MEDICAL PERIODICAL PRESS.

---

*Evidence afforded by the Microscope in a Case of Rape.* By A. F. Sawyer, M. D., San Francisco, California.

Last November, I was called to see a child of Mr. H., a little girl about five years of age, on account of suspected violence against her person. After the occurrence, the child came into the house looking very pallid, and complained to her mother of being sick, whilst her clothing was observed to be disordered and stained with blood.

On examination, the inside of the thighs, and the legs of the drawers, were found to be much marked with blood, and evidently some attempt had been made to remove the blood on the thighs by wiping. The external parts of the child were bruised and tumefied. The hymen had lost its natural pale color, and appeared highly congested. Its aperture was about the size of a crow-quill. There was no appearance of laceration in it, or in the surrounding textures. There was also a constant dribbling of urine during our examination.

All her clothing, consisting of Canton flannel drawers, a faded yellow petticoat, and a light outside frock, I caused to be sent to my office. I transferred, also, the little particles of dirt, noticed about her privates, especially in the folds of the integument, to a piece of fresh white paper, and afterward sponged them with a clean rag, and removed the piece of the sheet which had been moistened by the escape of the urine; all of which I preserved for future inquiry. I then visited the privy, where it was supposed the outrage had been committed. There was about half an ounce of fluid blood on the stool of the privy, and on the floor was a piece of newspaper smeared with blood, which had evidently been used for the purpose of wiping.

My attention was afterward directed to an examination of the party suspected of the crime, and who had been arrested in the afternoon of the same day. He wore, at the time of his arrest, an outer red flannel shirt, considerably soiled, covering a bluish-gray woolen shirt, which exhibited three or four small stains in front resembling blood; and a pair of drab colored woolen pants with two or three small suspicious-looking spots along the opening in front. His clothing was also sent to my office for subsequent examination.

From the considerable amount of hæmorrhage that had been noticed at the privy, and from the absence of distinct laceration of the hymen of the child, I thought it quite probable that the external parts of the accused would exhibit tearing—perhaps of the frænum—to account for the large amount of blood lost; and which, had it existed, would have supplied important evidence of his guilt. This was not the case, however. His yard was perfectly clean, without trace of blood, and bearing no marks of laceration or other injury.

By a microscopical examination of the spots on his clothing already referred to, those on the red shirt, more marked about the wrists, were found to have been caused by blood, as also the smaller stains on the gray shirt; and blood globules were distinctly visible in the field of the microscope, where a selection had been made from one of the stains of the pants that bore indications of attempts having been made to remove it by scraping or otherwise.

On examination of the piece of sheet which the child had wet, an abundance of blood globules was found, mixed with the cell-growth characteristic of semen. The rag used to bathe the privates of the child, exhibited the same appearances. The most important proofs were, however, detected in the microscopical examination of the particles of dirt that had been transferred to the piece of paper. In this was found, besides an abundance of blood globules and sperm cells, *fibres of wool mixed together, of distinct color, some of them being of a bright red, and others of a dirty indigo color, corresponding exactly with the wool fibres of the two shirts which the accused party wore at the time of his arrest.* The contrast of the colors was more marked with the reflected light of a candle, although perfectly distinct when observed by sunlight. After repeating these examinations, to remove every source of fallacy, the flannel petticoat of the child was examined, and its fibres preserved the characteristic color of the texture—a light yellow. This garment had lost its brightness of color by use.

The presence of sperm cells about the person of the child, clearly illustrated the cause of her injuries, and the detection of the wool-fibres from the same source was sufficient evidence to connect the party arrested with the commission of the crime. If the red fibres, or the indigo-colored fibres alone had been discovered, there might have been a wide margin for doubt; but the complete identification of the texture of both shirts afforded the strongest presumptive proof of the guilt of the prisoner, especially when taken in connection with the stains of blood detected on his clothing.

The large amount of blood which the child lost, withou any apparent laceration of the genital apparatus, is remarkable. It is clear to our mind that it came principally from tha hymen by the force of pressure, and most probably as a hæmorrhagic exudation similar to what is known to occur with other textures of the human body. It is to be borne in mind that the child was found in a weak and fainting condition. Notwithstanding the external bruises, sufficient force had been applied to create incontinence of urine. We had, also, the presence of blood globules in the urine which washed her parts as it escaped from her, and an examination of the urethra by a probe failed to detect any injury to this canal. The insides of the thighs were stained with blood, and apart from the known vascularity of the hymen, it was in a highly congested state at the period of our examination. There is no doubt that the hæmorrhage came from the child; and if from the child, in all probability from the hymen.

Otherwise, the principal testimony in the case rested with the child, and although given by a person of an age that would scarcely warrant its credi bility, seems to be entitled to some authority from its consistency. Besides conducting the police to the privy where the outrage was perpetrated, she selected the prisoner, after his arrest, as the guilty party, from a number of others who had purposely dressed themselves similarly to deceive her; and the same identification took place in the court room during the trial, when the prisoner was presented in citizen's dress.

The remaining testimony was but meagre. For some days previously, the prisoner had been playing with the child, who had received from him little presents of fruit, &c. No evidence existed to show that the prisoner had been seen in company with the child on the day this attempt at rape had been committed.

The defence asserted an alibi, which could not be sustained, and the jury found a verdict of guilty without leaving their seats.—*Boston Medical and Surgical Journal.*

